# Advancing Understanding of Just-in-Time States for Supporting Physical Activity (Project JustWalk JITAI): Protocol for a System ID Study of Just-in-Time Adaptive Interventions

**DOI:** 10.2196/52161

**Published:** 2023-09-26

**Authors:** Junghwan Park, Meelim Kim, Mohamed El Mistiri, Rachael Kha, Sarasij Banerjee, Lisa Gotzian, Guillaume Chevance, Daniel E Rivera, Predrag Klasnja, Eric Hekler

**Affiliations:** 1 Herbert Wertheim School of Public Health and Human Longevity Science University of California, San Diego La Jolla, CA United States; 2 Center for Wireless & Population Health Systems, Calit2’s Qualcomm Institute University of California, San Diego La Jolla, CA United States; 3 The Design Lab University of California, San Diego La Jolla, CA United States; 4 Ministry of Health and Welfare Korean National Government Sejong Republic of Korea; 5 Department of Preventive Medicine College of Medicine Yonsei University Seoul Republic of Korea; 6 Control Systems Engineering Laboratory, School for Engineering of Matter, Transport, and Energy Arizona State University Tempe, AZ United States; 7 Institute for Data, Systems, and Society Massachusetts Institute of Technology Cambridge, MA United States; 8 Lufthansa Industry Solutions Lufthansa Norderstedt Germany; 9 ISGlobal Barcelona Spain; 10 School of Information University of Michigan Ann Arbor, MI United States

**Keywords:** just-in-time adaptive intervention, JITAI, just-in-time, JIT, walking, physical activity, needs, opportunity, receptivity, mobile phone

## Abstract

**Background:**

Just-in-time adaptive interventions (JITAIs) are designed to provide support when individuals are receptive and can respond beneficially to the prompt. The notion of a just-in-time (JIT) state is critical for JITAIs. To date, JIT states have been formulated either in a largely data-driven way or based on theory alone. There is a need for an approach that enables rigorous theory testing and optimization of the JIT state concept.

**Objective:**

The purpose of this system ID experiment was to investigate JIT states empirically and enable the empirical optimization of a JITAI intended to increase physical activity (steps/d).

**Methods:**

We recruited physically inactive English-speaking adults aged ≥25 years who owned smartphones. Participants wore a Fitbit Versa 3 and used the study app for 270 days. The *JustWalk JITAI* project uses system ID methods to study JIT states. Specifically, provision of support systematically varied across different theoretically plausible operationalizations of JIT states to enable a more rigorous and systematic study of the concept. We experimentally varied 2 intervention components: notifications delivered up to 4 times per day designed to increase a person’s steps within the next 3 hours and suggested daily step goals. Notifications to walk were experimentally provided across varied operationalizations of JIT states accounting for need (ie, whether daily step goals were previously met or not), opportunity (ie, whether the next 3 h were a time window during which a person had previously walked), and receptivity (ie, a person previously walked after receiving notifications). Suggested daily step goals varied systematically within a range related to a person’s baseline level of steps per day (eg, 4000) until they met clinically meaningful targets (eg, averaging 8000 steps/d as the lower threshold across a cycle). A series of system ID estimation approaches will be used to analyze the data and obtain control-oriented dynamical models to study JIT states. The estimated models from all approaches will be contrasted, with the ultimate goal of guiding rigorous, replicable, empirical formulation and study of JIT states to inform a future JITAI.

**Results:**

As is common in system ID, we conducted a series of simulation studies to formulate the experiment. The results of our simulation studies illustrated the plausibility of this approach for generating informative and unique data for studying JIT states. The study began enrolling participants in June 2022, with a final enrollment of 48 participants. Data collection concluded in April 2023. Upon completion of the analyses, the results of this study are expected to be submitted for publication in the fourth quarter of 2023.

**Conclusions:**

This study will be the first empirical investigation of JIT states that uses system ID methods to inform the optimization of a scalable JITAI for physical activity.

**Trial Registration:**

ClinicalTrials.gov NCT05273437; https://clinicaltrials.gov/ct2/show/NCT05273437

**International Registered Report Identifier (IRRID):**

DERR1-10.2196/52161

## Introduction

### Background

There is great interest in the promise of just-in-time adaptive interventions (JITAIs) to support behavioral medicine. A JITAI is a behavioral intervention that is designed to (1) provide interventions and support during *just-in-time (JIT) states*, defined as times when a person would have a *need for support*, an *opportunity to act* in accordance with the support, and *be receptive* to support [1]; and (2) adapt over time to a person’s changing needs with the use of adaptation algorithms that strive toward enabling a person to meet clinically meaningful behavioral targets (eg, national recommendations for a given behavior) while accounting for the person’s current capabilities and constraints. Although there is a lot of interest in this type of intervention, more work is needed to advance the understanding of the foundational concepts implied by JITAIs, particularly the JIT state. The JIT state concept is inherently context dependent, dynamic, and likely to manifest differently for different people over time. Given this complexity, much of the work on JITAIs has focused on either creating interventions that are theory driven in terms of specifying JIT states according to a priori decision rules or through more data-driven approaches such as reinforcement learning. An important gap is the lack of a conceptual understanding of JIT states, which could be achieved by conducting rigorous theory-testing protocols designed to test dynamic hypotheses about JIT states.

The purpose of this paper is to describe a research protocol for a National Institutes of Health–funded Smart and Connected Health study (R01LM013107) explicitly designed to produce rigorous empirical evidence to study JIT states in the context of a physical activity (PA) JITAI. The structure of the paper is as follows. First, background information is provided about JITAIs that is necessary to understand the motivation for our system ID protocol. Next, a description of the system ID experimental protocol is provided, including the specific goals of the project, experimental design procedures, measurement approach, and analysis plan. Finally, a discussion and the implications of this work are offered in terms of future research on JITAIs.

### Improving Understanding of JIT States Within a Digital Health PA Intervention

There is convincing evidence indicating that PA is valuable for reducing the risk of colon, breast, endometrial, lung, and pancreatic cancers [2,3] and cardiovascular disease [4] and improving glycemic control [5]. With an aging population, step interventions could help prevent chronic diseases, reduce health care costs, and improve functional life years and quality of life [2-18]. The clinical guidelines for steps suggest 8000 steps per day for adults [19,20], but only one-third of this group meets the guidelines [21-31]. Across PA interventions for adults (eg, human-delivered and digital), results show increases of 496 steps per day achieved above baseline levels of 5000 steps per day, and even high-impact interventions peak at 1363 steps per day above baseline; both result in activity that is still below the guidelines. Even among interventions that produce an effect, maintenance is rarely measured, and when it is, it is not achieved by many participants [32-35]. Our long-term goal is to create a model-predictive controller-driven JITAI to increase walking that, we hypothesize, will be more effective than current PA interventions at supporting individuals in achieving and maintaining national guideline recommendations of at least 8000 steps per day averaged across a week [19,20].

Although there are many possible algorithmic approaches to achieve this, such as reinforcement learning [36] or recommender systems [37], this research effort is focused on the use of a model-predictive controller approach [38]. A model-predictive controller is an adaptation algorithm that uses time-series data from an N-of-1 unit [39,40] to support decisions over time in dynamic, often complex situations, such as dynamically providing support to a person to increase their PA. As the name implies, a central feature is a computational dynamical model, which is a series of mathematical equations that encode previous domain knowledge and include parameters that are estimated from data derived from each N-of-1 unit (ie, a person in this context), thus enabling the controller to account for individual differences in predictions. These computational models, similar to weather or climate forecasting models, enable rigorous simulations of a person’s likely responses to different types of support provided both now and in the future. For example, the model could be used to simulate a person’s response to the provision of a notification meant to nudge them to walk within the next 3 hours. The model would generate predictions on the likelihood that a person will walk after receiving the notification at each moment. In addition, the model can be used to simulate the potential synergistic or antagonistic effects that might occur because of different decisions that could be made. For example, using the model, predictions could be made on the potential diminishing effects of providing notifications over time owing to habituation or growing annoyance, particularly if notifications are sent when a person does not need them.

As this description implies, model-predictive controller-driven JITAIs are complex and, thus, are difficult to create using theory alone, which, historically, was the dominant way in which adaptive behavioral interventions were developed [41,42]. Instead, JITAIs require robust experimentation that enables empirical optimization of their elements, particularly the generation of the computational dynamical models that the controller uses to run simulations and, by extension, make dynamic decisions. As described in previous work [43], the empirical estimation and validation of dynamical models occur through system ID.

The system ID study described in this paper had 2 complementary but distinct aims. First, it aimed to gather empirical evidence on the concept of JIT states. By varying whether a notification is provided when the person is thought to be in a state of need, when they have an opportunity to walk, when they are thought to be receptive, or combinations of these 3 states, the experiment collected initial evidence for which aspects of the JIT state are most important for supporting the effectiveness of JIT interventions and whether this changes over time.

Second, the experiment was designed to collect the data needed to optimize a digital health intervention, *JustWalk JITAI*. The goal was to estimate and validate dynamical models that can be used to construct a model-predictive controller that can make decisions on the provision of support in given moments to achieve and sustain clinically meaningful PA targets. Prior work was used as a foundation to achieve these aims, particularly a dynamic model of social cognitive theory (SCT) that encapsulates domain knowledge about behavioral processes that influence PA [44-46]. The SCT models were refined using the newly collected data both to help us better understand JIT states and to develop models that can be incorporated into a multitimescale model-predictive controller.

## Methods

### Overview

#### Aims

The aim of this study was to conduct a system ID experiment to empirically assess the conceptual elements of a JIT state and estimate and validate dynamical computational models relevant to JIT states. This work was conducted to inform the development of a future model-predictive controller-driven JITAI. We had three broad hypotheses: (1) walking bout planning prompts that are provided when the system determines that individuals meet all 3 conditions of a JIT state—have a need, have an opportunity to walk, and are receptive to intervention notifications—will be more effective than when such prompts are provided when only some or none of those conditions are met; (2) idiographic computational models (ie, models developed by and for individual participants) can be produced that are effective at predicting contexts in which suggestions to go for a walk will be effective and how such suggestions and adaptive step goals combine to support a person in achieving both daily step goals and sustained engagement in steps per day; and (3) nomothetic analyses (ie, insights gleaned from data aggregated across participants) will reveal meaningful clusters for different types of contextual patterns and trajectories of change across participants. These clusters will enable the selection of initial dynamical model parameters and, by extension, the development of a generic semiphysical model that can be used as a starting point for new participants in a future model-predictive controller-driven JITAI. In aggregate, these results will also be used to empirically test the added value of previous domain knowledge, as encapsulated in previous computational models, for improving model prediction and response, with a basic autoregressive model with external input as a reference model that only accounts for previous domain knowledge in the form of variable selection but not the structure of their relationships.

#### Study Design Overview

Building on prior work, including the mobile health app *HeartSteps* (which was relabeled *JustWalk JITAI* for this study to continue on the control systems side of JITAI development) [47,48], we conducted a system ID experiment designed to study the theoretical concept of JIT states as a tool for fostering behavior change. The system ID experiment focused on two key intervention components: (1) notifications delivered up to 4 times per day designed to increase a person’s steps within the next 3 hours via either increased awareness of the urge to walk or via bout planning and (2) adaptive daily step goals. Both types of notifications prompting short walks within the next 3 hours were experimentally provided or not across variations of *need* (ie, whether daily step goals were previously met), *opportunity* (ie, the next 3 h are a time window when a person has an opportunity to walk based on their previous step data), and *receptivity* (ie, the person received <6 messages in the last 72 h and walked after notifications were sent). In addition, the suggested daily step goals also varied systematically across time rooted in a person’s baseline levels of steps per day (eg, 4000 steps) and gradually increasing until they met clinically meaningful targets (at least 8000 steps/d on average). Participants wore a Fitbit Versa 3 and used the study app for 270 days.

#### Technology

##### Wearable Sensor

The Fitbit Versa 3 is a wrist-worn, watch-style activity tracker that records participants’ steps and minutes of moderate or vigorous PA (*active minutes* in the language used by Fitbit) that the tracker detects based on accelerometer and heart rate data. The Fitbit tracker records the step and activity data, automatically synchronizes with the Fitbit server, and pushes to the *JustWalk JITAI* servers using Fitbit’s subscription application programming interface (API). It was recommended to participants to set the Fitbit to use one of the market-available watch faces with the following features: (1) always visible information about the current step count, daily step goal, and progress toward meeting the goal and (2) positive reinforcement (in the form of a fireworks display and vibrations) when the daily step goal is met. The list of watch faces that met these requirements was provided by the staff.

##### Mobile App

**Figure 1:**
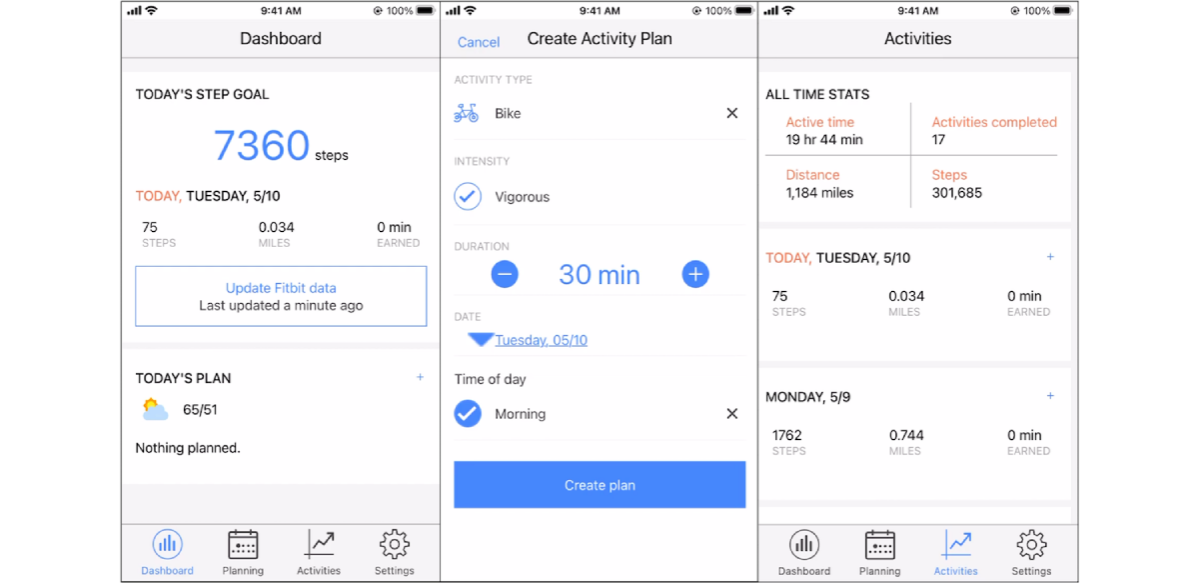
JustWalk JITAI app screenshots (left: app dashboard; center: planning tab; right: activity log tab).

**Figure 2:**
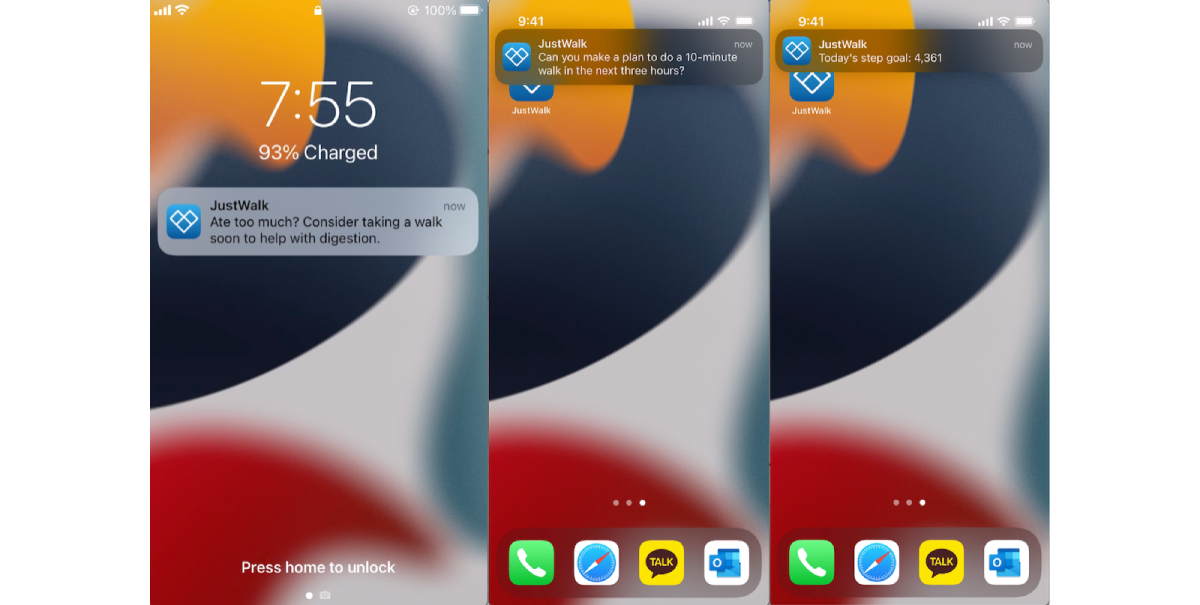
JustWalk JITAI app notification screenshot on the locked screen and background status.

**Figure 3:**
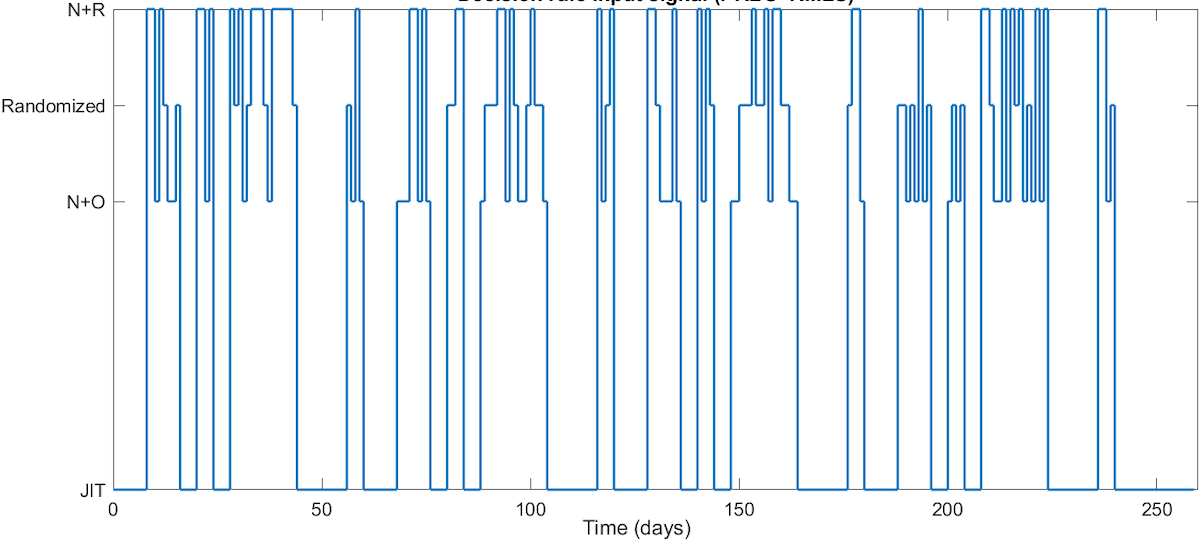
The designed decision rules signal for the walking notification component of the intervention in the time domain in the JustWalk JITAI study. Each level represents one of the decision rules. The 4 signal levels were obtained by superimposing a 3-level random multilevel sequence (RMLS) signal on the base pseudorandom binary sequence (PRBS) signal. JIT: just-in-time; N+O: need and opportunity; N+R: need and receptivity.

The *JustWalk JITAI* app contained (1) *pull* components that participants could access at any time by opening the *JustWalk JITAI* app (Figure 1), (2) *push* components that were sent to the participants as app notifications based on system-based rules (these were our key experimental manipulations and are described in the *Interventions and System ID Experimental Design* section and Figure 2), and (3) ecological momentary assessment (EMA) questions (described in the *Measures* section).

The *JustWalk JITAI* app consisted of 3 *pull components,* which were drawn from the *HeartSteps* app [48]. These were accessible through tabs along the bottom of the app screen (Figure 1; left):

1. *Dashboard*: the dashboard was the home screen of the app and was displayed whenever a participant entered the app or finished interacting with a *JustWalk JITAI* notification. The dashboard implemented 3 behavior change techniques [49,50]: self-monitoring, feedback on goal progress, and reminders of activity plans. At the center of the dashboard, a participant’s progress toward their daily step goal was shown as feedback. If participants created an activity plan for the day, the dashboard also displayed this plan for the participants.
2. *Planning*: through the planning tab (Figure 1; center), participants could identify when they would plan to exercise that week. The planning tool was designed to operationalize the behavior change technique implementation intentions [51,52] by enabling participants to identify when they would be active and for how long and identify a specific activity to engage in.
3. *Activity log* (Figure 1; right): participants could see an activity summary for the last 2 weeks, including steps walked and distance covered each day, as well as the types of activities that the Fitbit tracker detected automatically or the user logged manually (eg, running, hiking, walking, and yoga). In addition, the activity log displayed Fitbit-derived active minutes for each day. Finally, the tab displayed the participants’ all-time statistics—hours of active time, total distance walked, total counts of activities (detected or manually logged), and total number of steps recorded since the user started using the JustWalk JITAI system. These all-time statistics were intended to provide longer-time-frame feedback on what the participant accomplished over the duration of the study.

### Participant Procedures

#### Recruitment

Participants were recruited nationally using a noncontact approach. Participants were recruited mainly through university mailing lists and word of mouth. The targeted number of participants was 50, with a final fully enrolled sample of 48 as 2 participants never showed up to preintervention meetings.

#### Eligibility

Inclusion criteria were participants who (1) were aged ≥25 years; (2) were inactive, defined as engaging in <60 minutes per week of self-reported moderate-intensity PA; (3) owned either an iPhone with iOS 11 or above or an Android phone with Android 5.1 or above; (4) stated a commitment to follow study protocols, including regularly carrying a mobile phone, using the *JustWalk JITAI* app, answering phone-based questionnaires, and wearing the Fitbit Versa 3 activity tracker at least 8 hours a day; and (5) were fluent in English.

The exclusion criteria were participants who (1) were incapable of providing informed consent or (2) had a psychiatric disorder that limited their ability to follow the study protocol, including psychosis and dementia.

#### Screening, Informed Consent, and Onboarding Meeting

All participant interactions occurred remotely. Recruitment materials directed participants to the study website, which included a contact entry form. Upon completion, participants were automatically sent a link to an eligibility screener, which asked about age and PA levels (as reported using the Global Physical Activity Questionnaire [53] and the revised Physical Activity Readiness Questionnaire [54]). The staff reviewed the survey responses to confirm eligibility.

Eligible participants were offered time slots for an informed consent meeting. Ineligible participants were informed of their ineligibility.

During the informed consent meeting, the following activities took place: (1) the study was described in detail via a guided read-through of the consent form, (2) participants were provided with a list of reasons to and reasons not to take part in the study, (3) participants were invited to develop a list of their own pros and cons for taking part in the study, and (4) participants were given time to ask any questions they had. If participants verbally agreed, the study staff asked them to sign the consent form via DocuSign (DocuSign, Inc).

Consented participants were asked for a mailing address to send a Fitbit. Participants were also sent instructions on how to set up the Fitbit and the app via email. Once confirmation of delivery of the Fitbit was received by the staff, a follow-up email was sent to participants inviting them to pick a time slot for the preintervention meeting.

During the onboarding internet-based meeting, participants were instructed on the following topics: (1) instructions on how to install and use the Fitbit and study app, (2) information on the 10-day baseline, (3) information on what to expect after the 10-day baseline period, (4) direction to complete a baseline survey, and (5) instructional videos with corresponding notes about how to use and maintain the Fitbit (eg, strategies to keep it charged and reminders to clean it to reduce skin irritation). All meetings between the participants and the staff took place via Zoom (Zoom Video Communications), and the interviews took place within 2 weeks after the time slots were sent.

#### Incentives

Participants received the following incentives: (1) Fitbit Versa 3 (received at study enrollment; US $229 in value), (2) US $25 gift cards provided to them up to 3 times (US $75 in total) if they completed at least 80% of the daily EMA items within each 3-month period, and (3) US $25 gift cards if they attended an optional postintervention interview.

#### Study Timeline

During the 10-day baseline, participants were asked to engage in their normal level of steps or PA and always wear the Fitbit except while charging. No interventions were provided, and no EMA questions were asked during the baseline period. When participants opened the app, 10 circles were shown designating the number of days they had met the minimal wear time requirements (ie, 8 h/d). If the participant did not wear the Fitbit for at least 8 hours a day, the circles did not fill up. Once all 10 circles were filled, the app automatically transitioned to the intervention phase, displaying a dashboard.

In the intervention phase, all app features were delivered, including the 2 push intervention components (ie, walking prompts and adaptive suggested step goals) and the daily EMA questions. The participants also gained access to other parts of the *JustWalk JITAI* app such as activity logs and planning support. Participants were asked to interact with the app whenever it sent them notifications and were told that they could open the app at any time if they wanted to access pull components and found them useful. Total interaction time with the app from push interventions and EMA notifications does not exceed 10 minutes each day, but participants may choose to spend more time on the app accessing other features. The interactions participants were prompted to do occurred in response to four types of notifications: (1) daily step goal notifications, (2) walking suggestion notifications, (3) prompts to complete the daily EMA battery, and (4) experience sampling prompts (ie, if Fitbit detected an activity) to complete EMA items throughout the day and in relation to the notifications to either increase the urge to walk or plan (for details about EMA, see the *Measures* section).

### Interventions and System ID Experimental Design

#### System ID Overview

This study used a system ID approach to manipulate 2 intervention components experimentally: *walking suggestions* and *daily step goals*. To achieve the desired dynamics on the timescale of interest, we used 2 input signals, one for each of the 2 components. Although our study design enables traditional statistical analyses to examine the impact of intervention components on behavioral outcomes, that is not the primary focus of a system ID experiment. The primary goal of a system ID experiment is to estimate and validate dynamical computational models that are validated based on their ability to predict the future responses of each individual’s behavior across time. These aims are achieved by having different intervention components—suggestions to walk in the next 3 hours and adaptive goal setting—delivered at different timescales and orthogonally, that is, statistically independent of each other. Our approach is analogous to a within-person factorial experiment (and, indeed, can be treated as such with all relevant nomothetic statistics used on the developed data set, which the team plans to perform as secondary analyses). The critical difference is that, in system ID studies, the designed input signals achieve statistical independence through orthogonality as verified in the frequency domain. Orthogonality enables separate study and estimation of the dynamics and impact of each intervention component. One can think of frequencies as different repeating rhythms across time, such as the notion of a minute-by-minute, daily, or weekly frequency. The study was designed to ensure that the intervention signals were disambiguated across time (via delivery at different frequencies). This enables a rigorous independent study of dynamical responses to both intervention components within the same experiment and, indeed, within each person, both proximally (eg, immediate responses following intervention delivery) and distally (eg, continued or delayed effects up to several days after any notification).

Specifically, both signals are designed to follow the guidelines presented in the study by Rivera et al [55], in which equation 1 is highlighted to define the effective frequency range of the input signal based on a priori knowledge of the dominant system time constant.

Equation 1 is the equation used to define the effective frequency range of the input signals of the *JustWalk JITAI* study based on a priori knowledge:



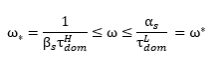




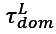
 and 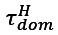 represent the higher and lower bounds for the estimated dominant time constant of the system, meaning the range in which signals relevant to walking would occur naturalistically. *α_s_* and *β_s_* dictate the input signal’s content of high and low frequency, respectively. The orthogonality of our intervention components was confirmed via the use of cross-correlation analysis (the appropriate approach for testing orthogonality via frequency domains) to the designed input signals.

#### Sample Size Considerations

The number of participants has little impact on the power estimate for system ID studies as system ID approaches mostly use dynamic models that consider individual-level changes over time [43]. Instead, different methods, such as study length and validation analysis, can be used to establish the equivalent notion of *power* [56]. A multisine signal’s *cycle*, or predetermined time interval, serves as the foundation for the power calculation [57]. By dividing the cycles into estimation and validation data sets, this type of design maximizes the signal-to-noise ratio and aids in the evaluation of model fits.

Previous research has demonstrated that 3 independently excited harmonics per cycle can achieve a sufficient excitation to deliver dynamically meaningful data in relation to daily frequencies. This can be performed with 3 sinusoids per cycle, resulting in a cycle that lasts at least 12 days [58]. Estimating and comprehending the multitimescale dynamics of behavior change are the goals of this effort. A total of 9 excited harmonics were revealed through simulations to be necessary to provide persistence of excitation across relevant frequencies [59]. The result is that each cycle lasts 26 days. From this, the final study length was set at 10 cycles (260 d).

#### Intervention Design

##### Overview

An overview of the *JustWalk JITAI* intervention elements is provided in Textbox 1.

**Summary of the JustWalk JITAI intervention elements**

**Wearable sensor**

- Fitbit Versa 3

**Mobile app**

- *HeartSteps* [47] (renamed *JustWalk JITAI*)

**Walking notifications**

- Walking notifications were pushed up to 4 times a day starting at the participant-set time (eg, 7 AM or 8 AM), with 3-h gaps between each possible notification (eg, 7 AM, 10 AM, 1 PM, and 4 PM as possible decision points for sending notifications).
- Notification texts were randomly chosen from a library of 50 messages that included 24 messages meant to inspire participants to plan a time when they would walk in the next 3 h and 26 messages meant to invite participants to become aware of internal urges that could inspire them to walk (Figure 2 and Multimedia Appendix 1).
- The experimentation setting used 4 just-in-time (JIT) definitions: (1) full JIT (need [N], opportunity [O], and receptivity [R]), (2) N+R, (3) N+O, and (4) random, with each element defined as follows:
  *N*: on track to meet the daily step goal accounting for time of day when assessed (eg, 50% of steps accrued halfway in a person’s day, using their self-selected start time as a reference and assuming 12-h windows)
  *O*: next 3-h time window was predicted to have an 80% likelihood that someone could take steps using a previously published algorithm [60]
  *R*: participant received <6 notifications and responded (ie, walked) to at least 50% of notifications sent to them within the previous 72 h

**Adaptive step goals**

- Each morning, participants were provided with a suggested daily step goal.
  The notification also included a single-item ecological momentary assessment whether it was helpful or not.
- Daily step goals were calculated using the following procedures:
  For the first cycle, median steps/d were used as a personalized reference to guide adaptive step goal suggestions. For cycle 1, a participant’s personalized reference (ie, median steps/d) was calculated from their 10-d baseline period excluding nonwear days.
  For all subsequent cycles, their personalized reference (ie, median steps/d) was calculated from the previous 26-d cycle (eg, cycle 2’s median steps/d were calculated using all step/d data from cycle 1 excluding nonwear days).
  Participants were provided with a step goal that ranged between their personalized reference (median steps/d) up to their personalized reference+4000 steps.
  A multisine signal design that ranged from 0 (personalized reference) to 1 (personalized reference+4000 steps) was used. For example, if a person’s median steps/d during the baseline period were 5000 steps/d, they would receive step goal suggestions between 5000 and 9000 steps/d.
  Maximum step goals were set at 12,000 steps/d, and minimum step goals were 2000 steps/d.

##### Walking Notifications

###### Overview

The first component of the *JustWalk JITAI* was notifications meant to inspire short (eg, ≥10 min) walking bouts within 3 hours after receiving the notification. This component had two variations that targeted different behavioral processes: (1) an invitation for a person to create a microimplementation intention on when, where, and how to fit in a ≥10-minute walk in the next 3 hours—we label these suggestions as *bout planning*—and (2) an invitation for a person to become aware of interoceptive experiences and signals (eg, stiff muscles and lethargy) that may inspire them to go for a walk—we refer to these messages as *cultivating an urge*. Both types of walking notifications were drawn from a library of 50 messages (n=24, 48% on bout planning and n=26, 52% on cultivating an urge). Walking notifications were provided in the form of push notifications from the *JustWalk JITAI* app. The notification could be sent 4 times a day (ie, decision points) starting at the user-defined *start of day* time, which was gathered during the onboarding processes. Starting from the participant’s self-described *start of the day*, the walking notification decision points occurred every 3 hours. For example, if a participant’s day started at 8 AM, their decision points would be 8 AM, 11 AM, 2 PM, and 5 PM. For each participant, on each day of the study at each of the 4 decision times, the *JustWalk JITAI* system decided whether to send a walking notification based on the system ID procedure described in the following section.

###### Operationalization of JIT States

JIT states were experimentally varied via the use of different rules to define a *JIT* state. By nudging participants when they were in *JIT* states, the hypothesis is that the effect of the walking notifications should increase while maintaining a low level of burden on participants, thus minimizing notification fatigue.

A JIT state has been previously conceptualized [1] as a state in which a person is receptive to support (eg, if a notification is sent, a person would appreciate receiving said notification) and has the opportunity to engage in the desired behavior (or vulnerability to a negative behavior). Building on this theoretical formulation, a third theoretical parameter was added: the need for intervention support. For example, if someone is already meeting their daily step goals, they likely do not need additional intervention prompts to walk. For the purposes of this study, JIT states were operationalized as follows:

1. *Need (N)*: a person is defined as in a state of need if they did not meet the previous day’s step goal (for the first decision point) or if they are not making steady progress toward that day’s goal (for all other decision points). Sufficient progress was defined as the goal prorated to the current time of day:

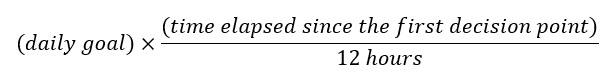

2. *Opportunity (O)*: a person is deemed to be in a state of opportunity when they can feasibly walk. To operationalize this, a predictive algorithm described in the study by Park et al [60] used a threshold of 80% probability that, within the next 3 hours, a person may walk. We used the high threshold of 80% so that even a slight nudge to walk could effectively achieve short-term behavior change (note: whether notification is needed at such a high moment of opportunity is a question that we will be able to study retrospectively).
3. *Receptivity (R)*: a person is deemed to be receptive when they have received ≤6 messages in the last 72 hours *and* have responded (ie, walked in the following 3 h) after ≥50% of the walking notifications sent in that period.

The operationalizations of these 3 facets of a JIT state allow us both to define a full JIT state—that is, when need, opportunity, and receptivity are *all* present—and to empirically test how different operationalizations of JIT states (eg, states when only some of these components are present) influence walking notification effectiveness. Specifically, daily decision rules were tested that embodied four different levels of being in a JIT state:

1. *Full JIT state*: need, opportunity, and receptivity are present.
2. Partial JIT state (2 forms): *N+O* (only need and opportunity are present) and *N+R* (only need and receptivity are present).
3. *Not in a JIT state (random)*: notifications are randomized each time at 50% probability.

How and when each of these rules for defining JIT states was varied experimentally is described in the following section.

###### Previous Observations and Theoretical Considerations That Guided Our Study Design to Test JIT States

This specific study design was created based on data from the original *HeartSteps* trial [47] followed by engaging with previous domain knowledge, including behavioral theory and our previously developed SCT dynamical model [61], to guide the final study design such that this study could provide robust data for supporting computational model testing.

Concerning previous observations, in the *HeartSteps* trial related to the notifications designed to inspire bouts of walking, 3 empirical observations guided our understanding of JIT states. First, as reported previously [47], notifications had a diminishing proximal impact on the total number of steps taken within the 30-minute window after the notification. These results indicated a theorized diminishing value-to-burden ratio of the prompts, namely, a dynamic concept that balances, for each instance, a person’s perceived value that they receive from an intervention compared with the perception of the level of burden of the intervention. This dynamic hypothesis, which conformed to the data, was that the value-to-burden ratio would diminish over time, with initial notifications being perceived as more valuable than burdensome; however, by the end of the study, this would shift toward a low or negative value in relation to the burden.

Second, it was observed that, if <2 notifications were sent on a given day, even later in the 6-week trial, then the bout notifications resulted in significantly improved steps taken within the 30-minute window after the notification. We interpreted this dynamic observation as representing a hypothesized *autorecovery* that could take place on a person’s value-to-burden ratio. One could think of this as analogous to a neuron. Once a neuron fires, if new signals come in, the neuron will not fire again until it has sufficient time to recover, but this recovery process is automatic. It was hypothesized that a similar dynamic takes place regarding notifications. Namely, if notifications are sent at a rate that is faster than a person’s autorecovery rate, habituation will set in and the notifications will be ignored (again, similar to a neuron not firing). If, in contrast, *sufficient time* has passed for autorecovery to take place (eg, such as a neuron re-establishing itself as ready for the next signal), then a notification sent would be more likely to be attended and reacted to by a participant. Previous data guided us to a population-based starting point of, on average, 2 notifications in a day, providing sufficient time for autorecovery. With that said, it was postulated that this autorecovery may vary among individuals. This study design enables us to study these individual differences in temporal responses.

Finally, it was observed that there was a trend in the daily timescale or frequency. Specifically, it was observed that there was an overall trend of increased steps per day over the 6-week intervention period. This third observation was translated into a hypothesized *internalization* process of the knowledge, skills, and practices that the intervention was meant to cultivate. This third dynamic hypothesis is the most critical target for designing an effective JIT intervention. Specifically, the goal is to create a JITAI that would enable a person to develop internalized knowledge, skills, and practices that could be maintained after the cessation of the intervention while accounting for the likely diminishing value-to-burden ratio and the need for *recovery* between notifications. This complex, interactive dynamic hypothesis, which postulates 3 different underlying dynamics that interact together, is what is primarily being studied in this experiment. Most critically, it was hypothesized that *internalization*, observed in the form of increases in steps per day in a time series, would take place more often when notifications or interventions were provided using JIT states compared with times when notifications were offered without taking account of JIT states.

###### System ID Experiment Design via Simulation Studies

With these empirical observations as a foundation, previous behavioral literature was reviewed to (1) look for previous domain knowledge that could be used to guide the understanding of these dynamics and (2) support us in better operationalizing the dynamic expectations we hypothesize to be observed, particularly if interventions could be provided consistently taking account of JIT states. A computational model was developed guided by these empirical observations and building on principles drawn from operant learning and cognitive science, which is described elsewhere [62]. A set of simulations was run to model anticipated responses to receiving PA notifications during positive and negative JIT states. A key focus of the simulation work was to determine whether the models could produce the dynamics observed in *HeartSteps*, described previously, and to guide the anticipated length of time needed to observe a possible overall step per day increase across days when notifications are repeatedly delivered during positive JIT states. In this context, a positive outcome was operationalized as a person taking at least 1000 steps (as a proxy for 10 min) within the 3-hour window after receiving a walking notification. Overall, it was hypothesized that a greater number of positive outcomes when using the full JIT state operationalization (N+O+R) would be observed, with increased overall steps per day occurring across days during those times (accumulative internalization). In contrast, it was hypothesized that a relatively steady steps per day response would occur during times when notifications were sent at random (which was a replication of the original *HeartSteps* study and intentionally did not consider JIT states; thus, it was hypothesized that the random signals would replicate the observations from the original study). A set of additional simulations was run based on the SCT model [61], with the results of the simulation reported elsewhere [59] to further refine our study design.

On the basis of the simulation results from both models, a system ID study was devised that experimentally varied the use of different definitions of JIT states but did so in a way that would enable the study of possible accumulation or degradation of the dynamic effects across days. Specifically, 4 days was set as the minimal length of days needed to observe the effects of successive full JIT rules. It was anticipated that stabilization to degradation of effects would start to occur within 1 day of sending non-JIT notifications based on our simulation studies. With that said, given the highly novel study design and limited robust empirical data to guide this subtle study of dynamics, longer periods were used, particularly for the full JIT state (N+O+R). In other words, the experiment compared decision rules that range from not trying to intervene in a JIT state to trying to intervene in a full JIT state over a sustained period that, based on simulation studies, would be sufficiently long to detect accumulative effects if they occurred.

This resulted in a categorical 4-level design. To construct this categorical 4-level input signal, a pseudorandom binary sequence (PRBS) was used (for full justification and details, see the study by El Mistiri et al [59]). This base signal compares JIT with some form of partial JIT or random (ie, non-JIT) states. To incorporate the exploratory examination of differences between JIT operationalizations, a random multilevel sequence was superimposed over one of the PRBS binary levels to compare the 2 incomplete JIT decision rules (N+O and N+R) with the randomly sent walking notifications [59]. The input signal design parameters for the PRBS were chosen as 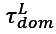
=3 days, 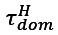
=3.5 days, *α_s_*=2*,* *β_s_*=2*,* which was done to cover the frequency range of interest based on the guidelines provided in equation 1. This resulted in a 60-day cycle with n*_r_*=4 shift registers and switching time *T_sw_*=4 days (Figure 3). This 60-day cycle enabled the team to (1) study the hypothesized dynamic, positive accumulative effect on steps within 3 hours of notification times, and steps per day when walking notifications were sent during theoretically defined JIT states; (2) compare these dynamics with the hypothesized dynamic degradation across days when walking notifications were delivered during partial or negative JIT states (ie, at random); and (3) as an exploratory aim, study if the dynamics vary across different JIT state operationalizations. In total, 4 cycles of a 60-day PRBS signal were generated to support both estimation and validation of the dynamical models that operationalized the hypothesized dynamics, which results in a 240-day period followed by a final period of full JIT state level to match the full study period (260 d), which was constrained by adaptive step goal cycles (26 d × 10 cycles).

**Figure 4:**
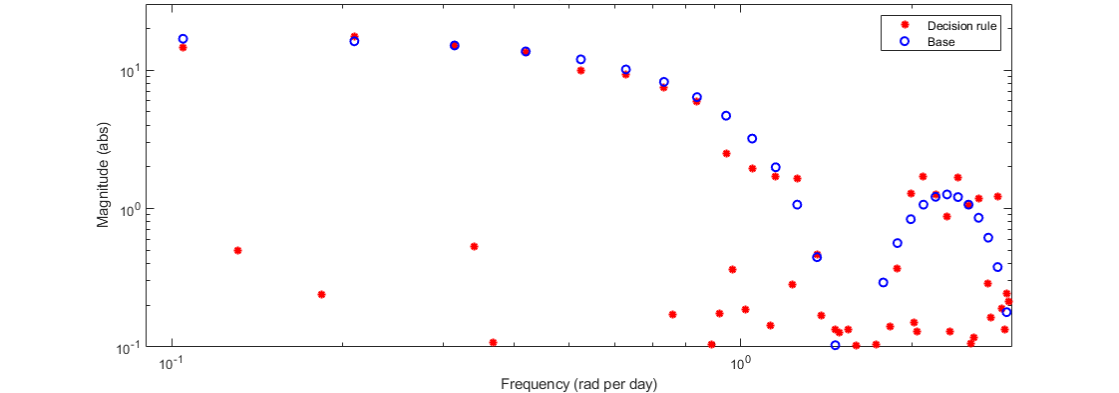
Spectral power density of the designed decision rule input signal of the JustWalk JITAI study. It is shown to determine whether sufficient excitation across key frequencies is established within the trial.

Figure 4 provides a visualization of the spectral power density as it relates to the walking notifications. This visualization provides insights into the degree to which the theorized dynamics will be appropriately excited, enabling the detection of effects if they occur across various frequencies and, thus, the study of the proposed dynamic hypotheses. The results suggest sufficient persistent excitation by the number of harmonics included in the effective frequency range between 0.14 and 0.67 rad per day. This frequency range, determined by the time constant guidelines in equation 1, ensures that the appropriate slow dynamics (ie, low frequencies) and fast dynamics (ie, high frequencies) of the system are captured.

##### Adaptive Step Goals Intervention Component

###### Overview

The adaptive daily step goal component follows a similar structure to that of a previous system ID experiment whereby participants were given a specific suggested step goal to target each day [63]. Similar to this previous design, a key consideration is to design a *cycle*, which is a deterministic, repeatable pattern that defines the provision of intervention options to an individual. Intervention options can be provisioned to mimic randomness via pseudorandom signal designs that achieve the valuable properties of randomness for causal inference while still being deterministic and, thus, repeatable. This provides valuable properties for a system ID experiment as it enables a more robust comparison between cycles (for more details, see prior work [38]). In this study, the same basic logic of prior work was followed, specifically, using a pseudorandom signal design that varied step goals between an *achievable* target up to a plausibly *ambitious* target (how goals are assigned each day is described in greater depth in prior work [59]).

The definition of an *achievable* step goal was personalized to each participant, which was labeled as a personalized reference, defined as a person’s median steps per day calculated from the previous 26-day cycle period [59]; note that, for the first cycle only, the personal reference was the 10-day-baseline period. Each morning, participants received a notification informing them of their targeted step goal for the day. The updated goal was also available to them on the *JustWalk* *JITAI* dashboard and was automatically synced by the *JustWalk* *JITAI* server to the participant’s Fitbit account so that the feedback on the Fitbit app and the participant’s Fitbit tracker always showed the correct goal progress each day. To further facilitate goal pursuit, participants were instructed to install a watch face providing the step goal number and a goal progress bar to enable always visible goal progress feedback. Fitbit’s native visual and haptic feedback was used when the participant completed the daily step goals (ie, fireworks animation and vibrations).

###### Experimental Manipulation: Input Signal Design

**Figure 5:**
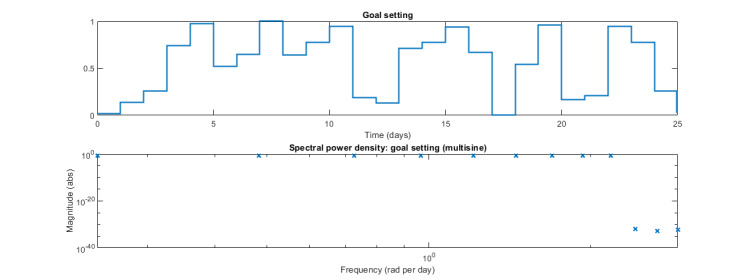
One cycle of the designed multisine input signal for the goal setting component of the JustWalk JITAI intervention in both the time (top) and frequency (bottom) domains. The multiplier factor varies between 0 and 1 over time. The spectral power density plot highlights the number of excited harmonics at the frequencies of interest.

To define a cycle for this component, a multisine signal was used. The input signal design parameters (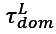
=1 day, 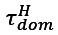
=2 days, *α_s_*=2*,* *β_s_*=2), as described in equation 1, were chosen based on the results from previous work and the simulation studies we conducted in preparation for staging this system ID experiment [59]. The design parameters result in a cycle length of 26 days, as shown in Figure 5. For each participant, a personalized realization of the multisine signal generates the daily goals throughout the 260-day intervention across 10 cycles by determining, for each day, the factor by which the 4000 steps per day range is multiplied and then added to the participant’s personalized reference (ie, median steps/d), as described in Textbox 1.

The effective frequency range of the signal is related to the design parameters through equation 1, which yields the persistence of excitation between ω_∗_=0.25 rad per day and ω^∗^=2 rad per day approximately for the designed multisine signal, as it is highlighted in the power spectrum of the signal shown in Figure 5. This showcases that the designed input signal for goal setting creates variability in the relevant dynamical ranges of interest.

### Measures

#### Baseline Survey

The baseline survey includes (1) demographic information, including age, height, weight, ethnicity, gender, race, marital status, household size, employment status, and level of education; (2) personal characteristics related to the study (ie, how the participants spend their time and information about their routine and their neighborhood); (3) self-perception (ie, personality [64] and perceived stress [65]); and (4) life habits [66] and PA [67] (ie, how they feel about PA, how they engage in exercise, how much they intend to exercise, and how they notice the effects of exercise).

#### Continuous Measurement of Activity and Heart Rate

Steps per minute and minute-level heart rate were measured using the Fitbit Versa 3, a wrist-worn, consumer-level activity tracker that uses triaxial accelerometry to measure movement. Further details are provided in the next subsection. Moderate or vigorous PA was measured in four ways: (1) automatically triggered objective measurement (the Fitbit Versa 3 automatically detects vigorous movement [68] if the activity is sufficiently vigorous and long), (2) manually initiated objective measurement (the Fitbit Versa 3 or the Fitbit app on the smartphone has an *activity* tab to log activity manually), (3) manual logging in the Fitbit app, and (4) manual logging on the study app using the *activity* tab (which was also populated with any reporting on the Fitbit app). The Fitbit assesses steps, PA intensity levels, energy expenditure, start or end time point and type of activity, distance traveled, and number of floors. Prior work shows that Fitbits likely underestimate heart rates and, by extension, total activity, but they do so reliably, thus establishing a meaningful within-person comparison [69], which is the focus of this study.

#### EMA Items

Psychological constructs and process variables were asked daily for inclusion in our targeted, dynamic models via EMA conducted at 7 PM local time. The EMA items included concepts of (1) self-efficacy for walking, (2) self-efficacy for problem-solving, (3) positive context for walking, (4) negative context for walking, (5) supportive routine, (6) drive to walk, (7) relationships supportive of walking, (8) interoceptive awareness of cues that could inspire walking (eg, stiffness and fatigue), (9) negative reinforcement of walking, (10) behavioral repertoire, and (11) typical supportiveness for walking. Detailed items are included in Multimedia Appendix 2. Items 1 to 6 were asked daily, items 7 to 10 were asked every 4 days to minimize the burden of responding, and item 11 was asked every day for the first week of each month (ie, 7 times/mo).

In total, 2 other types of EMA question items were sent. Triggered by completed PA, the participants were asked if they felt healthy, fatigued, energized, and discomfort. We also asked if the participants thought that they could meet the daily goal.

#### Weather

Daily weather data (current weather and weather forecasts) were gathered from the public weather database API [70]. No actual location GPS data were gathered; instead, a self-reported home zip code was used to gather weather data.

#### Postintervention Interviews

After the 260-day intervention, participants were asked to fill out a brief postintervention survey and were also given the option to participate in a postintervention interview. During the postintervention interviews, participants were asked about their overall reactions, including both positive and negative aspects of using the app and any suggestions to improve the intervention. The interviews were audio recorded and transcribed.

The participants were also given a choice to either stop the use of the *JustWalk JITAI* intervention or continue to use it, in which case the data past the study end date would not be used in the analyses.

### Treatment Fidelity Monitoring Procedures

#### Mobile App Use Logs

Mobile app use was recorded with time stamps for every page viewed in the *JustWalk JITAI* app, including opening notifications, opening the app, viewing pages within the app, and opening surveys. The one piece of information that could not be logged owing to operating system limitations was whether notifications (eg, walking notifications) were seen without being opened, such as when they automatically expanded on the iOS lock screen.

#### Monitoring the JustWalk JITAI Systems

The *JustWalk JITAI* server was automatically monitored every 10 minutes throughout the study period using a separate program to check for 5 performance and stability targets: the web server, the database server, the security firewall, the software framework for the server, and the Fitbit API. If the server stopped working or took too long to respond (>3000 ms), the program sent SMS text messages and emails to the study staff. The monitoring program was separately overseen by another program to ensure that monitoring was conducted properly. If any data operations failed (eg, if the Fitbit server was not responding), the study staff were immediately notified via email. If there was an error in the Fitbit data synchronization, when the data connection resumed, all the missing data were refetched to fill up any missing period.

#### Data Collection Monitoring

Data collection was monitored by the study staff on a weekly basis with automated visualizations to ensure that there were no technical errors that may compromise the study.

#### Study Adherence

Study adherence was monitored automatically using the *JustWalk JITAI* server. During the preintervention meetings, participants were asked to wear the Fitbit for a minimum of 8 hours a day, but it was suggested that they wear the Fitbit all day, even at night. Fitbit devices typically synchronize with the Fitbit server via the Fitbit phone app every 15 minutes. This synchronization stops if the Fitbit device runs out of battery or is not worn for several days. The *JustWalk JITAI* server regularly checks whether a participant’s device has stopped syncing with the Fitbit server, and if so, it sends an adherence SMS text message to the participant.

As an operationalized protocol, at 15 minutes before the first decision point for walking notifications (eg, 6:45 AM local time for most participants), if a participant had no Fitbit app synchronization records for 60 minutes (eg, since 5:45 AM local time for most participants), the server sent an automated adherence SMS text message including an approximate length of the period for which the updates were missed (eg, a few hours, a day, or a while) to invite participants to recharge and synchronize their Fitbit on the Fitbit app.

This approach helped avoid making the mistake of responding too immediately to the problem of data drops caused by accidental battery discharges. As it can be assumed that people do not carry their Fitbit charger around during the day, sending an immediate *charge it now* message when data updates stop during the day is unlikely to be an effective remedy. In addition, given that it only takes approximately 30 minutes to charge a Fitbit from fully depleted to lasting more than a day, it was assumed that sending these reminders before the start of the day would give participants a chance to charge their Fitbit.

### Modeling and Data Analysis

A series of system ID estimation approaches will be used to analyze the data and obtain control-oriented dynamical models to study JIT states. General data analysis will start with examining the cross-correlation of the data to verify the hypothesized structure of the system as operationalized via the computational models described previously. Nonparametric estimation methods such as correlational analysis and spectral analysis [71] will be used to obtain preliminary information about the responses of each individual (ie, time constants, gains, and orders). The knowledge gained from the nonparametric estimation methods will be used to obtain ideographic models through prediction error modeling approaches such as autoregressive with external input and output error [71] estimation and more elaborate gray box methods using the SCT model structure. In addition, the model-on-demand [72,73] estimation will be used to estimate more flexible models that address nonlinearities in the system. The estimated models from all approaches will be contrasted with one another, and the advantages or disadvantages of each will be assessed to inform future efforts.

### Ethical Considerations

The study was approved by the University of California, San Diego, institutional review board (protocol 800132) and was preregistered on ClinicalTrials.gov (NCT05273437).

## Results

### Simulations

The input signal design for the 2 intervention components in this study involved an iterative procedure that relied on a priori knowledge and simulation results for different types of anticipated participants to guide the efforts. The simulations were based on a dynamic SCT model derived from a fluid analogy, which provided the means to guide specific conditions for the JIT states considered in the decision rules to ensure that both the number of notifications sent per day and the overall number of notifications sent throughout the intervention were not burdensome for the participants. Furthermore, the simulation framework with diverse scenarios provided insights into the *ambitious yet achievable* range of adaptive goals provided in each goal setting cycle. A detailed account of the model used in the simulations, technical details of the designed input signals, and simulation results that guided the design are provided in the study by El Mistiri et al [59]. In this section, the results for a hypothetical adherent participant are presented to illustrate the dynamic nature by which the daily goals adapt to the participant’s performance, as well as the effectiveness of the JIT decision rules in limiting the provision of support only to times that are hypothesized to be beneficial.

**Figure 6:**
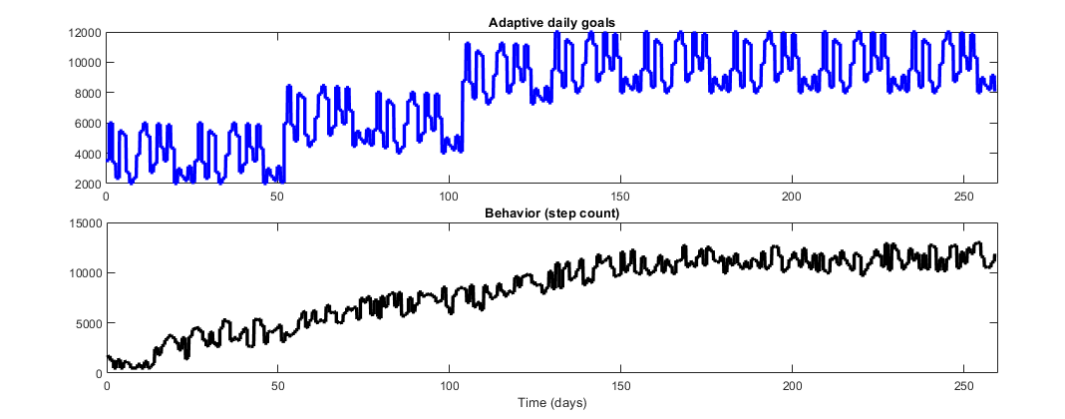
Simulation results illustrating the implementation of adaptive daily goals (top) in reaction to the performance of a hypothetical adherent participant in terms of daily step count (bottom) in the JustWalk JITAI study (adapted from the study by El Mistiri et al [59]).

Figure 6 [59] shows the implementation of the designed daily goal signal in a simulation setting. In this case, the goals in each cycle are adjusted to the performance of the participant in the previous cycle, as described in Textbox 1. Note that, in this case, a hypothetical adherent participant is capable of achieving the daily goals given to them in each cycle; consequently, the median of the participant’s performance increases, which leads to an increase in the goals provided in subsequent cycles. As a result, the daily goals gradually increase over the span of the intervention, from a low of 2000 steps per day in the first 2 cycles of the intervention (the first 52 d) to a high of 12,000 steps per day in the last 5 cycles. This simulation result illustrates that the input signal design for this component is working as intended by adapting the daily goals to each participant in a personalized manner while nudging the participant toward higher levels of PA through a combination of *ambitious* and *achievable* goals.

**Figure 7:**
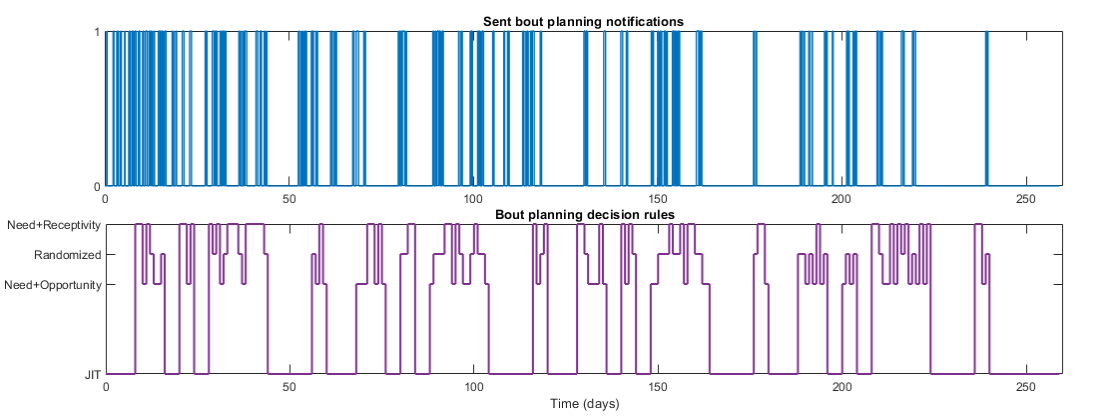
Simulation results for a hypothetical adherent participant illustrating the expected walking notifications (top) sent based on the designed decision rules signal (bottom) of the JustWalk JITAI study. For the sent notifications signal at the top, a 0 value implies that no notifications were sent at that decision point, whereas a value of 1 implies that a notification was sent (adapted from the study by El Mistiri et al [59]). JIT: just-in-time.

Figure 7 [59] shows the walking notification component of the intervention in the simulated scenario for the hypothetical adherent participant. As shown in the figure, the decision rules work as intended in terms of dictating the nature of the notifications sent to the participant. At the beginning of the intervention, when the participant does not achieve the daily goals (hence, the need condition of the decision rules is met), the number of notifications sent to the participant is high across all levels of the decision rules. Later in the intervention, as the participant adopts healthier behaviors and meets the daily goals, the number of walking notifications sent on a daily basis is significantly lower. Furthermore, note that, on days when the receptivity condition is considered, the number of notifications sent follows the notification budget mentioned in Textbox 1.

Finally, as the need condition is not met by the participant toward the end of the intervention, walking notifications are only sent on days with fully randomized notifications. This design allows for comparing the impact on the participant of fully randomized notifications with that of notifications that are guided by JIT state conditions that should make them more beneficial. From these simulation results, the rate at which notifications are sent (ie, notifications or decision point) on full JIT state days is the lowest at 0.084, followed by days of need and opportunity (N+O) conditions at 0.148 and need and receptivity (N+R) conditions at 0.176. The highest rate of notifications is observed on days with fully randomized walking notifications at 0.488.

### Recruitment

**Figure 8:**
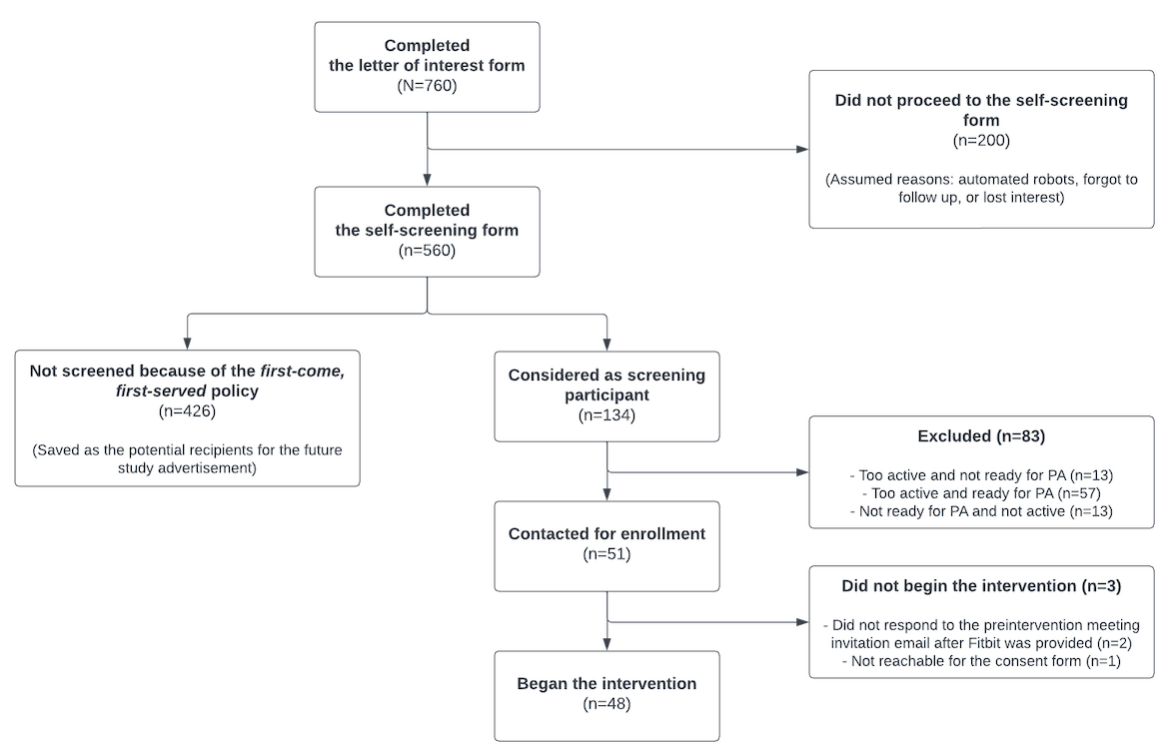
CONSORT (Consolidated Standards of Reporting Trials) recruitment diagram for the JustWalk JITAI study. PA: physical activity.

Enrollment began in March 2022 and ended in July 2022. In total, 761 potential participants submitted a letter of interest, and 48 (6.3%) were enrolled in the study. Figure 8 shows the CONSORT (Consolidated Standards of Reporting Trials) diagram [74]. The intervention was completed in April 2023. The data were gathered without major incidents. The source code for the server and the app is publicly available on the project’s GitHub repository [75].

## Discussion

This is a study protocol to investigate 3 JIT states (ie, need, opportunity, and receptivity) empirically and enable the empirical optimization of a JITAI intended to increase PA (steps/d) in adult populations with an inactive lifestyle.

There is well-documented evidence suggesting that digital health interventions to date have not lived up to their intended potential [76-78]. From issues of poor adherence; results that only produce limited effects; and questionable scalability, particularly among those with less access to digital devices, the potential of digital health interventions has not yet translated to a real-world impact [76,77,79]. A pathway for improving this is to focus on producing evidence directly targeting and seeking to improve the fundamental shortcomings of digital health interventions [77,78].

In this study, our primary focus was to rigorously examine the notion of JIT states. To date, JIT states used to formulate intervention decision rules have either been assumed to be correctly defined—typically based on guidance from behavioral theory—but not empirically tested or they have been derived using data-driven approaches such as reinforcement learning whereby previous domain knowledge and understanding is underemphasized and, instead, there is hope that useful insights about intervention timing will emerge from data collected in intervention studies [80]. Although we believe that both of these paths have merit, this study protocol offers a middle ground whereby previous domain knowledge is encapsulated into computational models, which, through simulation studies, can then be used to guide the careful generation of evidence that can test dynamic hypotheses about the nature of JIT states.

This is important as a JIT state, conceptually, is an inherently nonlinear causal phenomenon [81]. There is no one causal factor that makes a given moment a JIT state, but instead, it is a mixture of different factors such as time of day, a person’s current motivational levels, their relationships, recent experiences with the types of support given, and the degree to which support is well matched to a person’s current needs. Such factors combine at a given moment to influence the decision to engage—or not—in the target behavior. This study protocol recognizes the inherent nonlinear causal nature of the phenomenon under study and provides a rigorous approach to gathering the data needed to make progress in the context of such complexity. By varying whether a notification is provided when the person is thought to be in a state of need, when they are thought to have an opportunity to walk based on their personal historical step data, and when they are thought to be receptive, or combinations of these 3, the experiment will collect initial evidence for which aspects of the JIT state are most important for supporting the effectiveness of JITAIs and whether this changes over time. Using this information, particularly when linked with slow dynamic processes of change (ie, daily goal setting), the experiment produces data needed to empirically optimize a digital health intervention, *JustWalk JITAI*. Specifically, this work will result in individualized, empirically validated dynamical models that can be used to predict each individual’s response to the intervention options offered to them. These individualized or idiographic dynamical models can be applied to optimal personalized behavioral interventions through sophisticated control algorithms such as model-predictive control [43,82]. These model-predictive control–driven JITAIs could have the potential to work more effectively than previous digital health interventions.

The key limitations of this study stem from the high novelty of the overall experiment and its approach. To the best of the authors’ knowledge, no system ID experiment of this sophistication for studying human behavior has ever been conducted. On the basis of this, there was very little robust previous evidence and examples that we could draw upon to guide study design decisions. Although we did compensate for this by drawing on some relevant data (largely from our own work, as already described) and via a number of simulation studies, overall, there are potential risks and limitations to our approach. For example, given the novelty of this experiment, it is unknown how well the assumptions we used to guide the experiment will hold up. With this, it is unclear exactly how informative those data will be for studying JIT states. Second, given the novelty of this experiment, it was unclear what an appropriate sample size should be. This point is critical for determining the degree to which any patterns or insights gleaned about JIT states from this sample will be transportable to other populations or settings.

With that said, the primary focus of any system ID experiment is the study and articulation of computational models that are predictive and foster robust control decisions for each *system*. In this context, a *system* is a person. This is critical to note because, as flagged previously, the notion of *statistical power* as is used in the classic frequentist inferential statistics used most commonly by health scientists does not have any direct translation or use within system ID experiments. Indeed, the key focus of system ID experiments is to work within each system to gain a deep understanding of its dynamics. This focus makes sense particularly for a concept such as JIT states, which, definitionally, will likely manifest idiosyncratically. The critical question is not whether some general pattern of JIT states can be inferred but, instead, whether the same algorithmic development processes can be conducted ideographically and in a replicable and scalable fashion to enable the insights that the algorithm can produce to guide intervention decision-making. This is the primary focus of our work. Thus, the limitation is less one of *statistical power* and more akin to what arises with regard to the right training data sets for machine learning algorithms. It is unclear at this time what variations across people, places, and time could occur in real-world contexts that would render our approach nonfunctional. With a sample of only 48 participants, a key limitation is that we very likely did not have diversity across variations in people and places where this type of algorithm could be used to test the robustness of our approach. With that said, given the great novelty of our overall approach, we contend that this is an appropriate trade-off. Most critically, it is likely that, even in the sample of 48 participants, we will discover some individuals from whom we can create computational models that are informative and others from whom we cannot. That will be the type of initial data we could use to then develop more rigorous hypotheses about the transportability of our methods, which can then guide future experimentation.

Overall, this work could feasibly be a key step in filling the gap between the hope of digital tools and current realities in terms of limited long-term impact and engagement based on the evidence. Although this is all still quite hypothetical, this trial is a critical step in testing the potential benefits of this overall approach for intervention optimization.

## Appendix

Multimedia Appendix 1 Walking suggestion notification messages and their classification into 2 categories of messages.

Multimedia Appendix 2 Ecological momentary assessment items.

Multimedia Appendix 3 National Institutes of Health peer review report.

## Abbreviations

API: application programming interface
CONSORT: Consolidated Standards of Reporting Trials
EMA: ecological momentary assessment
JIT: just-in-time
JITAI: just-in-time adaptive intervention
PA: physical activity
PRBS: pseudorandom binary sequence
SCT: social cognitive theory

## Data Availability

The study materials and analysis code will be available through a public repository. The data set generated in this study will be available from the corresponding author upon reasonable request and with completion of the data user agreement.
