# Supplementary material for: Advancing Understanding of Just-in-Time States for Supporting Physical Activity (Project JustWalk JITAI): Protocol for a System ID Study of Just-in-Time Adaptive Interventions
Source: JMIR Res Protoc. 2023 Sep 26;12:e52161. doi: 10.2196/52161 (PMC10565629; doi:10.2196/52161)
Supplement: Multimedia Appendix 1 [file resprot_v12i1e52161_app1.docx]

**Multimedia Appendix 1.**

Walking suggestion notification messages and their classifications into two categories of messages

| **Messages** | **Category^1^** |
| --- | --- |
| How are you feeling? Think a 30-minute walk in the next three hours could make you feel better? | 1 |
| Feeling lonely? Could you walk and call a friend or ask a friend to walk with you in the next three hours? | 1 |
| Ate too much? Consider taking a walk soon to help with digestion. | 2 |
| Do you love podcasts? Any chance you could plan a walk and listen to a short podcast (even part of a long one) in the next 3 hours? | 1 |
| Feeling productive? If not, going for a short walk soon could feed your brain and help your focus and energy. | 2 |
| Feeling low? Even a 15-minute walk can often help. Can you take a walk in the next 3 hours? | 1 |
| Ate recently? Going for a walk is great for averting the food coma. | 2 |
| Feeling stressed? Do you think a quick walk soon might help? | 2 |
| Are you at a place with stairs? Would taking a few minutes to go up and down the stairs help you feel more energized? | 2 |
| Feeling anxious? Do you think a walk might help to clear your thoughts? | 2 |
| Need to get something? Is it nearby? If yes, consider walking there to get your activity and get something done at the same time. | 2 |
| Feeling sleepy? Taking a quick walk soon will help wake you up and feel better. | 2 |
| Having trouble sleeping at night? Do you think taking a walk in the next few hours may help you sleep better tonight? | 2 |
| Do you have a plan on when to be active today? If not, consider finding time in the next 3 hours. | 1 |
| Feeling bored? Would taking a walk soon help with reconnecting to your priorities? | 2 |
| Want to catch up with a friend? Text them to see if they could take a 10-minute walking break with you soon (phone or in-person). | 2 |
| Is there any point in the next three hours when you could take a break to take a 10-minute walk? | 1 |
| Feeling stiff? Would going for a walk soon help? | 2 |
| Is there a beautiful place nearby where you could go for a quick walk in the next few hours? | 1 |
| Feeling confused or conflicted about something? Would taking a quiet walk soon help clear your mind and give you some clarity? | 2 |
| Have you gotten enough activity today? If not, could you go for a short walk in the next two to three hours? | 1 |
| Want to live a long and healthy life? Being regularly active really helps. Could you take a walk in the next few hours? | 1 |
| How is your schedule looking today? Is there a free window in the next three hours when could walk? | 1 |
| Muscles feeling tight? If so, consider taking a walk in the next couple of hours to loosen up your body. | 2 |
| Craving a snack? Are you really hungry or just need to change things up? If the latter, consider taking a walk soon rather than eating. | 2 |
| Need to get something done but can't focus? A walk could help you clear your head so you can get things done more efficiently. | 2 |
| Are you feeling stuck about something? Consider taking a walk in the next few hours to clear your head and maybe get unstuck. | 2 |
| Can you make a plan to do a 10-minute walk in the next three hours? | 1 |
| Did you know regular activity is a key protective factor for dementia? Can you go for a walk in the next three hours? | 1 |
| Feeling frustrated? Would taking a short walk help you get some perspective? | 2 |
| Remember good being active can feel? Do you need that right now? if so, consider going for a walk soon. | 2 |
| Ate too much? Consider taking a walk to help you digest and not get sleepy. | 2 |
| Did you know regular activity helps you sleep better? Could you take a walk in the next few hours to help your sleep tonight? | 1 |
| Upset about something? Do you think taking a walk would help you calm down? | 2 |
| Can you see any gaps in your calendar in the next three hours when you may be able to take a 10+-minute walk? | 1 |
| Feeling like you can't get things done today? Consider clearing your head with a walk. | 2 |
| Trouble deciding on something? Consider letting your mind wander while walking. The answer just might come to you. | 2 |
| Feeling overwhelmed? Taking a walk may help you gain perspective and feel better. | 2 |
| Regular activity is an important part of keeping a healthy weight. Could you squeeze in a walk in the next few hours? | 1 |
| When the cravings come, consider short-circuiting them by getting away from food and going for a walk instead. | 2 |
| Feeling cold? Could a walk help warm you up? | 2 |
| Look at your calendar. See any times for a 10+ minute walk soon? | 1 |
| Look at your calendar. See any times for a 10+ minute walk soon? | 1 |
| Can you think of anything beautiful near you, like nice scenery or architecture? Do you think you could walk over there soon? | 1 |
| Any upcoming meetings? can any of them be walking meetings (including walking phone meetings)? | 1 |
| Are you going to be eating with a colleague, friend, or a family member in the next few hours? Could you take a post-meal walk together? | 1 |
| Do you have meetings in the next few hours? Could you end one 10 minutes early so you could go for a walk? | 1 |
| Will you be finishing a task in the next few hours? If so, could you go for a walk to reward yourself for a job well done (even if it's just answering email)? | 1 |
| Have you spent enough time with your family? How about taking a walk together (including a walking call) in the next few hours to catch up? | 1 |
| Thinking of watching TV soon? If so, could you replace a bit of that time with walking instead? We promise you you'll feel better. | 1 |

^1^Categories:

1) messages designed to inspire participants to plan a time when they would walk in the next 3 hours

2) messages designed to invite participants to become aware of internal urges that could inspire them to walk
