# Supplementary material for: Advancing Understanding of Just-in-Time States for Supporting Physical Activity (Project JustWalk JITAI): Protocol for a System ID Study of Just-in-Time Adaptive Interventions
Source: JMIR Res Protoc. 2023 Sep 26;12:e52161. doi: 10.2196/52161 (PMC10565629; doi:10.2196/52161)
Supplement: Multimedia Appendix 2 [file resprot_v12i1e52161_app2.docx]

**Multimedia Appendix 2.**

Ecological Momentary Assessment Items

**Daily EMA (asked at 7 pm local)**

| Index | Question Text | Frequency | Answering Options |
| --- | --- | --- | --- |
| 1 | Being active is **a top priority** tomorrow. | Daily | Likert (Not at all – Completely) |
| 2 | **Circumstances will help me** to walk tomorrow (e.g., nice weather, getting in nature, free time). | Daily | Likert (Not at all – Completely) |
| 3 | My **schedule makes it easy** to be active tomorrow. | Daily | Likert (Not at all – Completely) |
| 4 | I **expect obstacles** (e.g., no time, unsafe, poor weather) to being active tomorrow. | Daily | Likert (Not at all – Completely) |
| 5 | I know how to **solve any problems** to being active tomorrow. | Daily | Likert (Not at all – Completely) |
| 6 | I am confident I can **overcome obstacles** to being active tomorrow. | Daily | Likert (Not at all – Completely) |
| 7 | **No matter what**, I'm going to be active tomorrow. | Daily | Likert (Not at all – Completely) |
| 8-1 | In general, my **friends help me** to be active. | Daily, but one of the three question items was asked per day | Likert (Not at all – Completely) |
| 8-2 | I regularly feel **urges to** be active. |  | Likert (Not at all – Completely) |
| 8-3 | I am active because it **helps me feel better** (e.g., reduce stress, stiffness, or fatigue). |  | Likert (Not at all – Completely) |
| 9 | My **typical Sunday includes being active.** | Daily for the first week of each month | Likert (Not at all – Completely) |

(Bold texts were shown as bold on the app)

**Activity Triggered EMA (asked within 15 minutes when a physical activity is detected)**

| Index | Question Text | Frequency | Answering Options |
| --- | --- | --- | --- |
| 1 | Are you **feeling healthy** now? agile, fit, limber, strong... | Activity triggered | Likert  (Not at all – Completely) |
| 2 | Are you **feeling fatigued** now? tired, exhausted... | Activity triggered | Likert  (Not at all – Completely) |
| 3 | Are you **feeling energized** now? awake, lively, vigor... | Activity triggered | Likert  (Not at all – Completely) |
| 4 | Are you **feeling discomfort** now? tired, aches, sweat... | Activity triggered | Likert  (Not at all – Completely) |

(Bold texts were shown as bold on the app)

Daily Step Goal EMA (asked individually set morning time (i.e., start of a day))

| Index | Question Text | Frequency | Answering Options |
| --- | --- | --- | --- |
| 1 | Today’s step goal: x,xxx  I think I can meet today’s goal | Local time (start of day) | No / Maybe / Yes |
