# Supplementary material for: Advancing Understanding of Just-in-Time States for Supporting Physical Activity (Project JustWalk JITAI): Protocol for a System ID Study of Just-in-Time Adaptive Interventions
Source: JMIR Res Protoc. 2023 Sep 26;12:e52161. doi: 10.2196/52161 (PMC10565629; doi:10.2196/52161)
Supplement: Multimedia Appendix 3 [file resprot_v12i1e52161_app3.pdf]

SUMMARY STATEMENT

PROGRAM CONTACT: (Privileged Communication) ***Release Date:*** 08/13/2018

Revised Date:

Application Number: 1 R01 LM013107-01

Principal Investigator

HEKLER, ERIC B

Applicant Organization: Regent of the University of California

Review Group: ZRG1 IMM-F (50)
Center for Scientific Review Special Emphasis Panel
PAR-18-949: NSF/NIH Smart and Connected Health (SCH)

Meeting Date: 08/07/2018 ***RFA/PA:*** PAR18-949
Council: OCT 2018
Requested Start: 10/01/2018

Project Title: SCH: Control Systems Engineering for Counteracting Notification Fatigue: An Examination of Health Behavior Change
SRG Action: Impact Score:24 Percentile:8 #
Next Steps: Visit https://grants.nih.gov/grants/next_steps.htm
Human Subjects: 10-No human subjects involved
Animal Subjects: 10-No live vertebrate animals involved for competing appl.

Project Year	Direct Costs Requested	Estimated Total Cost
1	[redacted]	[redacted]
2		
3		
4		
TOTAL	[redacted]	[redacted]

ADMINISTRATIVE BUDGET NOTE: The budget shown is the requested budget and has not been adjusted to reflect any recommendations made by reviewers. If an award is planned, the costs will be calculated by Institute grants management staff based on the recommendations outlined below in the COMMITTEE BUDGET RECOMMENDATIONS section.

SCIENTIFIC REVIEW OFFICER'S NOTES

RESUME AND SUMMARY OF DISCUSSION: This project involves the application of control modeling to walking/stepping behavior, extending previous work on the approach conducted in a theoretical social cognitive framework to the domain of physical activity. Specifically, the model will dictate just-in-time appropriate feedback to be delivered to participants via a mobile application to prompt bouts of walking/stepping when participants are amenable, most able to do so, and when it is feasible contextually. The significance of the project relates to its potential to develop timely alerts that are more optimal to signal walking/stepping and avert alert fatigue whereby individuals ignore alerts over time. The work proceeds in three phases, a) further consideration of the existing control model for the social cognitive framework and an alternative AutoRegressive with external input (ARX) model, b) an extensive micro-experiment aimed at specifying and tuning the model jointly for the likelihood of readiness to step and the scope of intervention features, and c) new exploration and simulation running off several existing and newly formulated models to find as many other capacities in the planned monitoring and feedback approach as possible. Strengths of the study include the existing dataset upon which this work builds, the investigators strong track record of collaboration, and the potential for this work to inform many other disciplines and practical applications. While some panel members consider the experimental design to be focused and based upon clear rationale, others noted insufficient or unclear discussion provided for certain study rationale, design, and methodology as well as a lack of certain experimental controls. Additional noted weaknesses, not shared to the same degree by the full panel, center on affective features such as stress and mood which are weakly covered and, on the projects' computational complexity. Overall; however, considerable enthusiasm was generated among the panel for the novel and creative high impact approach to the development of predictive models for inferring optimal internal and external conditions under which smartphone notifications have potential to result in increased walking activity and to be extended to other domains.

Intellectual Merit:

Strengths

- Panelists saw the work as novel and valuable for physical activity research. It constitutes a creative extension and combination of complementary lines of control and physical activity research of the investigators.
- Panelists were uniformly very excited about this proposal as a well-articulated project embedded in a strong programmatic line of research.
- Particularly interesting is the possibility of deploying a control model that functions simultaneously for the two timescales of data, momentary and daily in order to drive feedback to prompt walking bouts.
- Further innovation has already proceeded in the form of research methods, where the use of a randomized micro-experiments as a model validation approach at the nexus of model optimization and participant response could constitute a crucial best practice demonstration for future work in feedback-driven optimal control applications.

Weaknesses

- Some minor issues came up in discussions as opportunities for refinements in the work plan. For example:
- The compensation plan could possibly interfere with assessing the step feedback.
- Increase clarity of the physiologic and affective measures.
- Consider increasing the effect size target, which is on the low side for intensive within-

person data.
- Consider making the final exploratory phase overlap with the micro-experiments stage and making it more succinct.

Broader Impacts:
Strengths
- As a truly SMART system tailored to both user and contextual data, advancement of the science of physical activity support is very likely and the systems to be developed are likely to cross over to other health behaviors. This means the potential for board scientific impacts is substantial.

Weaknesses
- The dissemination plan could be improved by increasing engineering and computer science outlets.

Solicitation-Specific Criteria:
- **Infrastructure Planning, Software Sharing, and Data Management Plan:** It is unclear which software algorithms will be shared via open-source venues, and which venues they will be. An adequate data management plan is included.
- **Collaboration, Management, and Coordination (including letters of collaboration):** The plan is reasonable for exchange of information and coordination among investigators. However, given the three separate and geographically distant locations of the investigators, special attention to logistics is strongly recommended. In addition, possible additional concurrent engineering of stage II and III is highly recommended for consideration at study outset at the beginning of stage II.

Evaluation Plan: The evaluation plan contains many specifics, but it can be difficult to glean the design and methods. Factors in the 2x2 factorial experiment are difficult to determine from information provided. Also, the nature of experimental controls are not readily apparent from information provided.

Human Subjects: Protections appropriate and well-developed.

Budget: Appropriate.

Results from prior NSF and/or NIH support (if applicable): The proposed project builds upon NSF and NIH funded research.

Summary Statement: Developing and advancing dynamical modeling to decrease the ubiquitous alert fatigue as an approach to improve health behavior is an important and significant goal. The investigators bring a substantial body of work and experience from which to build upon in achieving aims outlined in the proposal. The final phase could be clarified, especially in how it integrates with the micro-randomization phase.

DESCRIPTION (provided by applicant): A wide range of technologies, such as smartphones, wearables (e.g., Fitbit, Apple Watch), and medical devices use alerts to inspire actions of users. Potentially useful alerts come at the cost of alert fatigue whereby individuals ignore alerts over time. For example, several physical activity interventions use alerts to inspire activity; notifications work initially but with diminished efficacy over time. Ignoring alerts is problematic in a variety of domains. For example, notification fatigue reduces the potency of

interventions (e.g., notifications to inspire walking) and can be a safety risk in other areas such as in hospitals where notification fatigue can lead providers to ignore safety alerts (e.g., cross-drug interaction) provided by the electronic medical record. There is a need for novel solutions for reducing alert fatigue. Location, digital traces, and other data enable inference of states when a person would desire/need alerts, henceforth labeled just-in-time states, but more advanced analytics are needed. For example, a suggestion to walk (e.g., SMS saying, "Want to go for a walk?") may only produce the desired outcome when a person's state (e.g., low stress) and context (e.g., no meetings, nice weather) align such that the person appreciates the notification (what we label receptivity) and can act on it (what we label opportunity). Estimating the likelihood that a given moment is a just-in-time state requires not only data but also an approach to manage the multivariate, dynamic, idiosyncratic, and multi-timescale nature of the problem. Returning to the walking example, stress, weather, and location change dynamically with each influencing the likelihood that a notification will inspire walking. In our work, results suggest idiosyncrasy in the factors that predict steps: some people walk more when stressed, others less, and still others are not influenced by stress. Further, just-in-time notifications cannot be viewed in a vacuum and, instead, are often part of a more long-term process, such as sustained engagement in a health behavior, thus making it a multi-timescale problem.
The purpose of this work is to develop a just-in-time state estimation strategy and to stage a multitimescale controller for walking as a concrete use-case of a control systems approach to counteract alert fatigue. Previous work (IIS-1449751) translated control systems techniques to ones suitable for just-in time behavioral interventions and ongoing work (HeartSteps) provides ample data to stage our approach and a technology platform to evaluate it. Secondary analyses of our HeartSteps datasets will be conducted using a control engineering approach for inferring just-in-time states for walking. These models will be linked with a previously created daily timescale model and a multi-timescale hybrid model predictive controller to support decision-making about when to send notifications and personalized daily step goals to support accumulation of walking bouts into meaningful change, which will be evaluated in a n=50 cohort study.

Intellectual Merit (provided by applicant): Intellectual contributions include: 1) a state estimation framework for defining just-in-time states for alerts that can manage the multivariate, dynamic, and idiosyncratic nature of the problem and that can be used with longer timescale models; and 2) a staged multi-timescale controller that can be used to support dynamic decision-making on when to send notifications to facilitate meaningful change.

Broader Impact (provided by applicant): As alarm fatigue is pervasive, the broader impact is enormous. It could be a boon for interventions involving accumulative behaviors such as physical activity, diet, medication adherence, smoking, and alcohol consumption, which, combined, explain ~40% of variance in health outcomes. A just-in-time state estimator could increase the likelihood that an intervention will foster behavior change by providing support when it is desired and needed, including within medical systems (e.g., safety alerts in electronic medical records). The analytic approach could be extended to other domains that require multivariate, dynamic, idiosyncratic, and multi-timescale alarm management such as medical devices (e.g., artificial pancreas systems, pacemakers). Reach will be extended via training of students in a transdisciplinary team and releasing our validation dataset for other alarm management work.

Critique 1:

Intellectual Merit:

Strengths

- The proposed activity involves the development of predictive models for inferring optimal internal and external conditions under which smartphone notifications will most likely result in increased walking activity among inactive and overweight adults. The investigators will develop a Just-In-Time (JIT) state estimator, validate the estimator "in the wild", and model its predictive ability via simulation. Targeting physical activity through step goals is important given the impact of sedentary behavior on common chronic conditions and general health.
- Use of a robust existing dataset to develop a state estimator for inferring opportunity to engage in physical activity behavior or planning.
- The plan to carry out the proposed activities is based on a strong rationale with clear milestones for success.
- Findings from the proposed activities have the potential to contribute significantly to multiple disciplines (health, engineering, computer sciences).

Weaknesses

- It is unclear how routines are built into the study design and conceptualization of JIT "opportunity", particularly in regards to planning activities. There may be similar simple and intuitive variables that would explain a lot of the variance in receptivity to notifications, such as whether the person works and opportunities for walking in a given job.
- Information on the Multi-arm Bandit approach is limited. It is unclear how this approach takes into account the potential impact of increased likelihood of a given message option on targeted outcomes. How data from this approach will be analyzed is also unclear.
- It is unclear how Phase 3 of the project, simulation of model predictive control specification, fits with the rest of the proposed activities, or how it will lead to an "integrated decision strategy" for JIT interventions.

Broader Impacts:
Strengths

- The knowledge gained from this project has the potential to be informative for a variety of disciplines, from behavioral science and healthcare to human computer interaction and control systems engineering.
- The investigators have a track record of dissemination of research, and there are plans for open-source code and anonymized data sharing.

Weaknesses

- No efforts for integrating underrepresented groups into the project are explicitly stated.
- It is not clear how educational goals or student training will be achieved in the project.

Evaluation Plan: The proposed activities include an evaluation of the JIT state estimator "in the wild" among a proposed 50 sedentary and overweight participants. Following a 2-week baseline period, participants will undergo 96 days in a micro-randomization trial, followed by 80 days of the Multi-armed Bandit approach. Outcome measures will be complete interaction with the notification and steps. This evaluation plan seems adequate to test the proposed aims.

Results from prior NSF and/or NIH support (if applicable): Prior NSF and NIH funded work lays the groundwork for this proposal. Data collected in this work identified individualized predictors of physical activity among overweight individuals. Previous work

provided evidence of effectiveness in regard to behavior change (steps) and usability of the mobile app used in the proposed activity.

Additional Solicitation-Specific Review Criteria:

- **Infrastructure Planning, Software Sharing, and Data Management:** All data associated with the project, including software and other products, will be tracked, stored, and published.
- **Collaboration, Management, and Coordination (including letters of collaboration):** The collaborators all have unique expertise that are relevant for the proposed activities. They have a history of collaboration and have had ongoing meetings and contact for several years on related projects. Each PI will lead components of the project that are most aligned with their expertise. In addition to regular teleconference meetings, the PIs will meet at regular professional conferences.

Human Subjects: Potential risks to human subjects and safeguards to protect against these risks are clearly outlined. Plans for recruitment and retention are adequate. Inclusion of participant groups is justified.

Budget: Appropriate.

Summary Statement: Responsiveness to the SCH Solicitation: The proposed activity is a multi-disciplinary collaboration that includes personnel from fields highly relevant for the SCH solicitation (i.e., clinical psychology, engineering, computer science). The outcomes of the proposed activity have high potential for making a large impact on health. There is limited integration of educational opportunities as part of this project. The proposed activities have high potential for impact on the fields of healthcare, informatics, and engineering. The investigators are well qualified to carry out these activities and have a strong track record of successful collaboration. The techniques proposed to identify optimal conditions under which responsiveness to notifications will be most effective are novel, creative, and can change the way Just-In-Time interventions are delivered across a variety of health behaviors. While some sections lack the clarity required to thoroughly evaluate promise of the planned activities (e.g., simulation of model predictive control specification), the primary strengths of the majority of the proposal place this application in the Excellent range.

Critique 2:

Intellectual Merit:
Strengths

- Using smartphone and wearable device technology with dynamic modeling, collaborative investigators propose a project to develop timely alerts that are more optimal to signal walking and avert alert fatigue.
- Advances modeling to estimate behavioral states for receptiveness to triggering a targeted health behavior (i.e., steps).
- Builds upon existing data and algorithms from previous work by investigators. The PI and co-investigators bring rich experience and expertise, with the proposed project building and improving upon novel approaches for estimating behavioral states via data-driven and dynamic modeling.

- The just-in-time modeling is grounded in Social Cognitive Theory and empirical work.

Weaknesses

- It would be beneficial to consider constructs beyond the Social Cognitive Theory that influence decision-making and engagement in health behaviors (e.g., mood, perceived benefits, motivation).
- More information on how heart rate data would be used as an indicator of stress would be beneficial. How would this data be used and analyzed (e.g., change equations, control for baseline levels)? The impact and innovation of the project would be enhanced by including more information on biometrics and how this would be used in estimating behavioral state via wearable technology. There is much potential in this area, which does not seem to be realized or fully addressed in the current proposal.
- Study compensation is tied to engaging in the targeted health behavior, which introduces potential bias for studying the efficacy of the model and intervention for increasing walking behavior. More discussion of the justification and rationale for linking the behavior with reward points is warranted.

Broader Impacts:

Strengths

- Targets the health behavior of walking, which is impactful for health promotion, prevention, and quality of life.
- The just-in-time state modeling, if successful, has potential applications to other health behaviors (e.g., diet) and could be used to decrease alert fatigue that is common in healthcare settings.

Weaknesses:

- More information on student involvement would be beneficial.

Evaluation Plan: The evaluation plan contains many specifics, but it can be difficult to glean the design and methods. Factors in the 2x2 factorial experiment are difficult to determine from information provided. Also, experimental controls are not readily apparent from information provided.

Results from prior NSF and/or NIH support (if applicable): The proposed project builds upon NSF and NIH funded research.

Additional Solicitation-Specific Review Criteria:

- **Infrastructure Planning, Software Sharing, and Data Management:** It is unclear whether software algorithms will be shared via open-source venues. An adequate data management plan is included.
- **Collaboration, Management, and Coordination (including letters of collaboration):** The plan is reasonable for exchange of information and coordination among investigators.

Human Subjects: Protections appropriate and well-developed.

Budget: Appropriate.

Summary Statement: Responsiveness to the SCH Solicitation: Developing and

advancing dynamic modeling to decrease the ubiquitous alert fatigue as an approach to improve health behavior is an important and significant goal. The PI and co-investigators bring a substantial body of work and experience from which to build upon in achieving aims outlined in the proposal. More explicit and cohesive information on the evaluation study design and methodology, with justification, is needed to fairly evaluate the proposal.

Critique 3:

Intellectual Merit:
Strengths

- Although the proposal does not use this phrase explicitly, this proposal involves one way of delivering a computationally driven health guidance system for physical activity. Previous (NSF and NIH) work involved demonstrating the tractability of control systems type models in relation to social-cognitive theory and applications, as well as development of a battery of tools for step behavior. This proposal focuses on integrating and enhancing those components with a focus on multi-input systems and with attention to delivery of feedback based on system features such as accumulation of steps, fatigue, etc. The proposal is couched in a programmatic line of research leading to large-scale trials, but this project remains in the basic science space.
- Very experienced and productive team that has worked together on multiple aspects of an integrated research program.
- Persuasive arguments that deriving information from lower time scales (momentary) could inform just-in-time guidance at the daily level, which can in turn be in various ways updated to guide further feedback.
- Useful combination of assessing receptiveness to feedback and identifying the kind of feedback needed.

Weaknesses

- The fundamental innovation could be seen to have already been realized in previous work and these refinements are tangibly in the application space, albeit with considerable additional complexity to be reckoned with and scientific goals that are crucial to understanding how to automate feedback.
- The planned microexperiments are potentially overplayed. It is not clear to me that adherence and compliance issues may not impair some modeling at the group level. That said, some participants will complete the 96 days and that may be sufficient to pursue the scientific goals to drive the JIT system. I am not certain that the particular targets can be varied effectively enough to see differentiation with total N = 50. I might have been more persuaded by a simpler demonstration of one most favored set of target levels.
- The third aim seems misplaced to me. This simulation-based work could in some ways precede project phase 2, and it certainly could overlap with it for more than one month. There may be system level constraints that could be understood in advance of the experiment that could better inform target levels.
- To the extent that the social cognitive model is retained, current SCH model is only one such model, one that is weaker on affective features such as stress and mood. Additional theoretical and instrumentation work is needed in addition to the planned largely computational work.

Broader Impacts: This project is likely to impact formulation of rather highly tooled interventions. Because of the computational complexity, and the need to tune the models in a very labor-intensive way to individual cases, it is not clear to me that fields with more behavioral complexity than just steps will quickly adopt this. On the other hand, combined with previous scholarship, it represents a throughput opportunity to demonstrate how this model can deliver just-in-time feedback to participants based on their data streams. This is part of the current direction of digital health, and it is likely that, with some lag, that other applications will grow into readiness for this approach. In an ironic sense, I believe that the timescale difference for the propagation of this work mirrors the study, whereas momentary behaviors drive daily feedback, it is probably years of study to epochs of scientific innovation ahead. However, the rapid proliferation of both health technology and scientific innovation may reduce the length of this lag. So, the strength of addressing complex questions and behaviors with complex tools may relate to a weakness in speed of likely dissemination.

Additional Solicitation-Specific Review Criteria: This proposal is highly in line with the solicitation. The concept of alerts is fundamentally a 'smart' technology problem with connectivity demands.

Summary Statement: In summary, I believe that this is valuable work by an accomplished team that should proceed. My reservations are that the complexity and 'technical novelty' may make project less impactful than previous ones without strong attention to what should be a primary goal of demonstrating key aspects of how to determine alert status for health behaviors. The overly complex microexperimental framework and its locus on the project schedule, combined with possible dissemination lags mitigate my enthusiasm further.

Critique 4:

Intellectual Merit: The proposal tackles an important area around alert fatigue from an interesting perspective, looking at Just in Time states, dealing with contextual information that impacts alert receptivity. The approach is also multi-time scale, examining acute and long-term patterns of behavior. The emphasis on tackling "teachable moments", translating a human systems view to a machine learning context also was an interesting way to think about this work. The experimental design is robust, focused, believable, and systematically thought out. It is one of the best experimental designs I've seen for this type of work. The power analysis is based on needed observations, rather than simply number of research subjects, addressing both the traditional social science concerns, but grounding these in a big data context. I appreciated that the authors were direct about the issues that come up with in situ work, describing the loss of a fitbit device with one former participant. These real-world problems offer some of the best assurance that the team has deployed with real people and dealt with the human factors issues that come up. Detailed descriptions of efforts to improve model fit are focused on human systems as well.

Broader Impacts: The issue of alert fatigue is applicable across a wide range of domains, including several key areas in healthcare, but also in many other situations involving human actors - ranging from military, to human systems controllers in engineering or industrial contexts. The authors explicitly point out where innovations could have important impacts and clearly demonstrate that their approach will live beyond this project. Healthcare impacts suggested include a range of behavioral interventions with patients, in hospital alerts for physicians and

healthcare staff that go beyond simple threshold levels and take into account expert input on personalized alert threshold setting. The authors are clear that their use case is complex enough that learnings in this setting take into account issues that may be found in very different case uses.

Additional Solicitation-Specific Review Criteria: The project looks to integrate data and analytical approaches from several projects, with retrospective coding on prior collected data to begin approaching questions raised by this proposal. These retrospective codings also try to take into account human behavioral elements not captured in initial work to improve context awareness. The experimental proposal described in 5.2 includes both micro-randomization and multi-arm bandit using these updates approaches to make improvements to an existing HeartSteps system, which has been successfully used for prior funded studies. The authors expect to collect 10s of thousands of observations over about 50 participants. Meaningful clinical outcomes are also included, e.g. sitting/walking, planning, presence of just in time intervention, etc. These data integration and analysis strategies are both sophisticated and grounded in real world human behaviors. Such combinations suggest that the team has a detailed understanding of how humans actually function, and that the contextual information they are gathering paints a realistic picture of the decisions humans take when encountering a behavioral alert.

Summary Statement: This is an exceedingly well thought out proposal. The even incremental gains in the domain of alerting/alert fatigue hold important promise within population health, behavioral health, and health care more broadly. Further, learnings from this project will likely inform many other disciplines and practical applications where attending to alerts is important but degrades over time. The use case is believable, and sufficiently complex to allow findings to be translated to other domains readily. The project incorporates data, analytic techniques, and tools from multiple inter-related projects. The experimental approach is emphasized (in part because the analysis relies on a robust experimental set up). The experimental design is focused, with clear outcome variables and predictors. The power analysis equally takes into account traditional social science and big data considerations. Further, the authors clearly have thought through problem areas that could arise and have a back-up plan in the event elements of their experiments are not as efficient in producing usable findings as expected. This proposal was extremely well structured. There are no significant weaknesses. The one comment I have is already anticipated in plans for future work, which is to expand to one or more other use cases to ensure generalizability and to gain insight from the differences that arise with these additional contexts. The proposal was a pleasure to read.

THE FOLLOWING RESUME SECTIONS WERE PREPARED BY THE SCIENTIFIC REVIEW OFFICER TO SUMMARIZE THE OUTCOME OF DISCUSSIONS OF THE REVIEW COMMITTEE ON THE FOLLOWING ISSUES:

PROTECTION OF HUMAN SUBJECTS: ACCEPTABLE

INCLUSION OF WOMEN PLAN: ACCEPTABLE

INCLUSION OF MINORITIES PLAN: ACCEPTABLE

INCLUSION OF CHILDREN PLAN: ACCEPTABLE

COMMITTEE BUDGET RECOMMENDATIONS: The budget was recommended as requested.

SCIENTIFIC REVIEW OFFICER'S NOTES: The Resume and Summary of discussion is essentially identical to the NSF panel summary written for this application, with minor modifications. The roster contains the names of all the reviewers participating in all eight of the Smart and Connected Health subpanel meetings.

Footnotes for 1 R01 LM013107-01; PI Name: Hekler, Eric B

Ad hoc or special section application percentiled against "Total CSR" base.

NIH has modified its policy regarding the receipt of resubmissions (amended applications). See Guide Notice NOT-OD-14-074 at http://grants.nih.gov/grants/guide/notice-files/NOT-OD-14-074.html. The impact/priority score is calculated after discussion of an application by averaging the overall scores (1-9) given by all voting reviewers on the committee and multiplying by 10. The criterion scores are submitted prior to the meeting by the individual reviewers assigned to an application, and are not discussed specifically at the review meeting or calculated into the overall impact score. Some applications also receive a percentile ranking. For details on the review process, see http://grants.nih.gov/grants/peer_review_process.htm#scoring.
